# Tackling barriers to COVID-19 vaccine uptake in London: a mixed-methods evaluation

**DOI:** 10.1093/pubmed/fdac038

**Published:** 2022-04-04

**Authors:** Kristoffer Halvorsrud, Jenny Shand, Leonora G Weil, Andrew Hutchings, Ana Zuriaga, Dane Satterthwaite, Jennifer L Y Yip, Cyril Eshareturi, Julie Billett, Ann Hepworth, Rakesh Dodhia, Ellen C Schwartz, Rachel Penniston, Emma Mordaunt, Sophie Bulmer, Helen Barratt, John Illingworth, Joanna Inskip, Fran Bury, Deborah Jenkins, Sandra Mounier-Jack, Rosalind Raine

**Affiliations:** Department of Applied Health Research, University College London (UCL), London WC1E 7HB, UK; UCLPartners, London W1T 7HA, UK; Department of Clinical, Education & Health Psychology, UCL, London WC1E 6BT, UK; UK Health Security Agency, London SE1 8UG, UK; Department of Health Services Research and Policy, London School of Hygiene and Tropical Medicine, London WC1E 7HT, UK; UK Health Security Agency, London SE1 8UG, UK; NHS England and NHS Improvement London, London SE1 6LH, UK; Office for Health Improvement and Disparities, London region, London SW1H 0EU, UK; Public Health England London, London SE1 8UG, UK; Office for Health Improvement and Disparities, London region, London SW1H 0EU, UK; NHS England and NHS Improvement London, London SE1 6LH, UK; NHS England London Shared Service, London SE1 6LH, UK; Association of Directors of Public Health, London EC4Y 0HA, UK; UCLPartners, London W1T 7HA, UK; UCLPartners, London W1T 7HA, UK; UCLPartners, London W1T 7HA, UK; Department of Applied Health Research, University College London (UCL), London WC1E 7HB, UK; UCLPartners, London W1T 7HA, UK; Office for Health Improvement and Disparities, London region, London SW1H 0EU, UK; NHS England and NHS Improvement London, London SE1 6LH, UK; Royal Free London NHS Foundation Trust, London NW3 2QG, UK; Department of Global Health and Development, London School of Hygiene and Tropical Medicine, London WC1E 7HT, UK; Department of Applied Health Research, University College London (UCL), London WC1E 7HB, UK

**Keywords:** COVID-19, ethnicity, inequalities, London, vaccination

## Abstract

**Background:**

In response to the COVID-19 pandemic, the first vaccine was administered in December 2020 in England. However, vaccination uptake has historically been lower in London than in other English regions.

**Methods:**

Mixed-methods: This comprised an analysis of cumulative percentage uptake across London between 8 December 2020 and 6 June 2021 by vaccine priority cohorts and ethnicity. We also undertook thematic analyses of uptake barriers, interventions to tackle these and key learning from a qualitative survey of 27 London local authority representatives, vaccine plans from London’s five Integrated Care Systems and interviews with 38 London system representatives.

**Results:**

Vaccine uptake was lower in Black ethnic (57–65% uptake) compared with the White British group (90% uptake). Trust was a critical issue, including mistrust in the vaccine itself and in authorities administering or promoting it. The balance between putative costs and benefits of vaccination created uptake barriers for zero-hour and shift workers. Intensive, targeted and ‘hyper-local’ initiatives, which sustained community relationships and were not constrained by administrative boundaries, helped tackle these barriers.

**Conclusions:**

The success of the national vaccination programme depended on conceding local autonomy, investing in responsive and long-term partnerships to engender trust through in-depth understanding of communities’ beliefs.

## Introduction

The COVID-19 pandemic reached the UK in late January 2020. Vaccines to protect against infection and severe disease have been rolled out at an unprecedented speed in high-income countries, with England being the first to establish a vaccination programme commencing on 8 December 2020.^1^^,^^2^

For vaccinations to work, uptake must be high across the country and among all social groups.^3^ However, London has historically lower vaccine uptake than other English regions.^4–7^ This is explained by multiple factors: uptake tends to be lower among ethnic minority groups,^8^ with London having the most ethnically diverse region and largest Black and Mixed ethnicity population in England.^9^ Vaccination rates are also lower in deprived areas; London includes 7 of the top 10 local authorities (LAs) for income deprivation among older people in England.^10^ High population turnover and outdated general practitioner lists to estimate denominators also hinder uptake.^11–13^

Given the need to achieve rapid coverage for resumption of ‘normal life’, we analysed uptake of the first vaccination dose across London during the programme’s first 6 months (i.e. between 8 December 2020–6 June 2021) by vaccine priority cohorts (Joint Committee on Vaccination and Immunisation (JCVI), Supplementary Material 1) and ethnicity. We then explored system/public health staff’s perceived barriers to vaccination for their London residents and interventions at any levels to address these, including learning for future immunization programmes.

## Methods

We comprehensively mapped vaccination uptake across London, supplemented by in-depth qualitative data to explain the quantitative results. We also explored initiatives to address uptake barriers.

### Quantitative methods

We extracted data from the National Immunisation Management Service about uptake of the first vaccine dose among individuals registered with the National Health Service (NHS) in London, between 8 December 2020–6 June 2021, and among total eligible population. We also obtained data on uptake by cohort and ethnic group, in the same period, from the NHS Foundry system.

Trends by weekly cumulative percentage uptake were examined for Cohorts 1–12.^1^^,^^14^ As Cohorts 10–12 opened between 13 April–8 June 2021 (i.e. near or after data collection ended), trends for 16 Office for National Statistics ethnicity categories^15^ were examined for Cohorts 1–9 only.

### Qualitative methods

We gathered qualitative data from multiple sources, as explained below.

#### Survey and Integrated Care System plans

A survey was piloted and refined with 12 public health staff and 5 patient and public involvement representatives before a SurveyMonkey link was emailed to London’s 32 LA’s Directors of Public Health (DsPH) or their nominated representatives. It comprised mainly open-ended questions on vaccination barriers, interventions and success factors within LAs (Supplementary Material 2). The survey ran between 24 March and 12 April 2021. Email reminders were sent where needed.

We also examined the COVID-19 Vaccination Delivery Plans that London’s five Integrated Care Systems (ICSs) submitted to NHS England/Improvement London performance team on 13 March 2021 for priority Cohorts 1–4 and on 23 March 2021 for Cohorts 5–9.

#### Semi-structured interviews

Subsequently, semi-structured interviews were conducted with a purposively selected sample, including survey respondents, to provide more in-depth insights. Respondents consisted of DsPH, ICS public health leads and decision-makers from NHS England and Public Health England in London.

Semi-structured interviews (~30 minutes) were completed on 24 May–4 June 2021 via Microsoft Teams due to COVID-19 restrictions. Topics included: strategies that had worked; challenges and learning for the COVID-19 vaccine programme and beyond. Interviews were recorded and transcribed verbatim.

#### Thematic analysis

All qualitative data were coded inductively—rather than restricting to a predetermined theoretical framework, the study subjects’ original wording thematically framed the analysis.^16^

The survey was independently coded by four coders (JS, EM, RP, CE), allowing wider discussion to refine themes.^16^ A review of similarity/divergences in coding (EM) and adjudication of unresolvable decisions (KH) were incorporated. Documentary thematic analysis^16^ was then used to analyse ICS plans against the survey domains. Preliminary themes helped develop the interview topic guide (Supplementary Material 3). Themes were further refined inductively, with interview transcripts checked by JS, EM and coded by RP, SB. Group discussion resolved disagreements.

We report summaries of number of LAs representing subthemes (combined from survey and ICS plans), supplemented by in-depth insights (or quotes) from the interviews.

Microsoft Excel was used to manage the qualitative coding/analysis.

### Ethics

This is a service evaluation, with no ethic permissions needed.^17^ However, participants provided consent and, complying with General Data Protection Regulation, identifying details were removed.

**Fig. 1:**
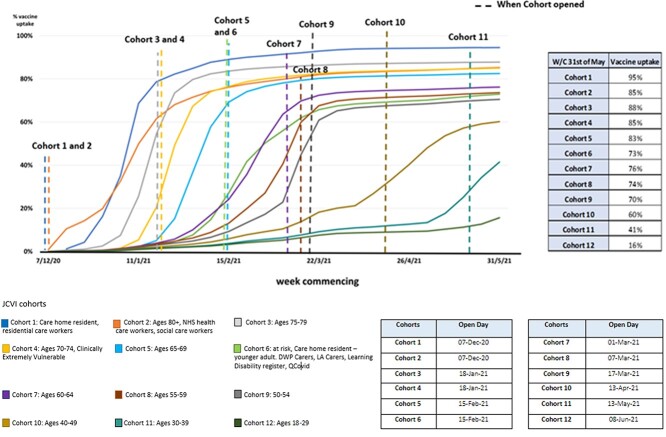
Cumulative percentage of first dose vaccine uptake by JCVI Cohorts 1–12 in London (8 December 2020–6 June 2021), including the date that each cohort opened.

## Results

### Quantitative findings

The 8 310 472 individuals in JCVI Cohorts 1–12 (3 656 050 in Cohorts 1–9) were included in the analysis of uptake of the first vaccination dose between 8 December 2020 and 6 June 2021.

Figure 1 demonstrates rapid uptake when vaccination opened for Cohorts 1 and 2 and by Cohorts 3–9 before they were officially eligible. Cohorts 1–9 then level off with limited further uptake recorded from April 2021; uptake at 6 June 2021 was between 95 (Cohort 1) and 70% (Cohort 9). In Cohorts 10–12, uptake also began before officially eligible. By the end of data collection, there was some evidence that uptake was levelling off in Cohort 10 but not in Cohorts 11 and 12. However, Cohort 11 was only officially eligible for vaccination 3 weeks before our data collection period ended, while Cohort 12 did not officially open until 2 days after the end of data collection.

**Fig. 2:**
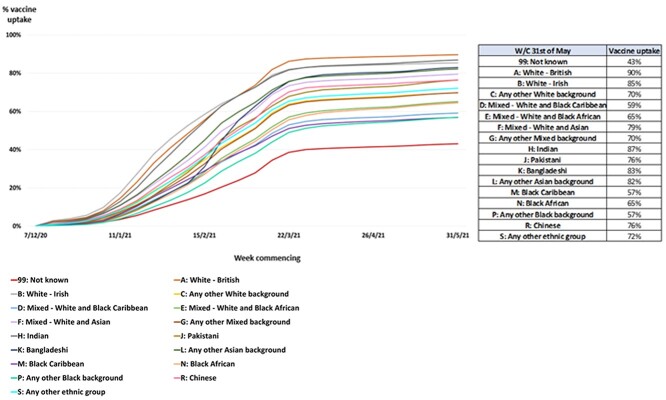
Cumulative percentage of first dose vaccine uptake in London by ethnicity for JCVI Cohorts 1–9 combined.

Uptake varied by ethnicity (Fig. 2). Uptake was highest for White British (90%), Indian (87%), White Irish (85%), Bangladeshi (83%) and any other Asian (82%) populations. Uptake was lower in Mixed White and Black Caribbean (59%), Black Caribbean (57%), any other Black background (57%) and those with unknown ethnicity (43%). Apart from a comparatively rapid increase in February–March 2021 in the Bangladeshi population, the relative differences in uptake were similar for all ethnic groups between January–June 2021.

### Qualitative analysis

Twenty-seven out of 32 LAs responded to the survey; the analysed vaccine plans covered London’s 5 ICSs, while 38 system representatives were interviewed (representing 21 LAs, 5 ICSs and 7 individuals from London Region). Further 11 representatives across all ICSs were also invited for an interview, but they declined the offer.

We divide our results into two overarching themes: demand for the vaccine (associated with ‘Vaccine Hesitancy’) and vaccine supply (associated with ‘Access’ to vaccination). Within both themes, we report barriers, interventions (including success factors) and future lessons.

### Demand for vaccination (vaccine hesitancy)

#### Barriers

The three major demand side barriers were: (i) lack of trust in government institutions; (ii) lack of trust in information and (iii) belief that costs outweighed benefits.

##### (i) Lack of trust in government institutions

Thirteen LAs stated that public health and vaccination staff had reported that, for ethnic minority residents, the most frequent rationale for vaccine hesitancy was distrust of government institutions. This was due to ‘inequalities, deep rooted racism and historic injustices’, which led to concerns of adverse repercussions for residents with a ‘lack of ID or proof of address, immigration status’ (LA respondents). Although the level of mistrust was ‘difficult to quantify or fully scope’ (LA respondent), respondents described how residents’ mistrust was so deeply engrained that:

‘[i]t’s not something that’s going to go away, and we’re not going to … shift that those … Those that mistrust for quite some time. So I think we have to be honest with ourselves … why people, you know, find it difficult to say, yeah, I’ll get my vaccination’ (ICS respondent)

Direct ramifications for uptake were reported:

‘[T]here have been issues around confidence and trust in the vaccine and different levels of trust have impacted on differential uptake in vaccine in different groups (eg linked to ethnicity, disability)’ (LA respondent)

##### (ii) Lack of trust in information

Ten LAs also reported lack of trust in information, particularly by ethnic minority communities and women of childbearing ages, emanating mostly from specific social media/online platforms. A lack of information and concern surrounding infertility was highlighted as contributing to vaccine hesitancy for women of childbearing ages and their families:

‘When we talked to them [resident women], they said “look, our fathers are frightened that no one will marry us if we take the vaccine”’ (LA respondent)

##### (iii) Belief that costs outweighed benefits

Zero-hour contract workers were reportedly anxious about taking unpaid time off to get vaccinated and to recover from any side effects (*n* = 5 LAs). Shift workers, such as domiciliary care staff, had similar concerns, exacerbated by travelling far from work to mass vaccination sites.

#### Interventions

**Fig. 3:**
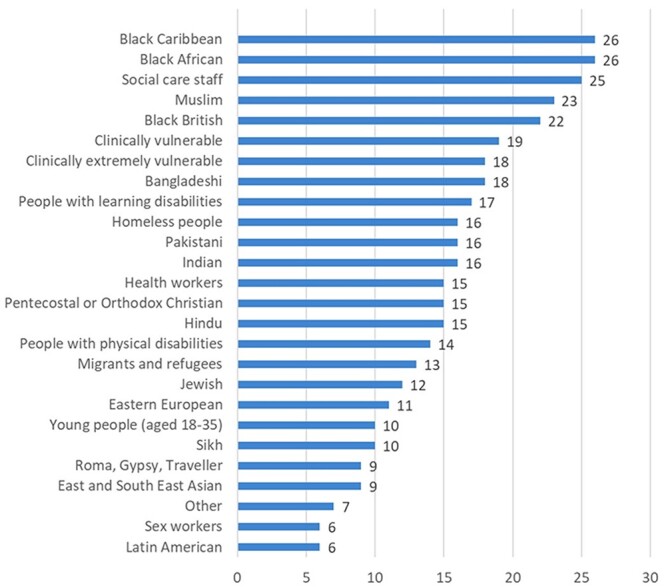
Number of LAs (*n* = 27) reporting activities to address demand side barriers (‘Vaccine Hesitancy’) among specific communities.

Figure 3 shows the different extent to which vaccine hesitant groups were targeted, with Black Caribbean and Black African groups targeted most (26 out of 27 LAs, respectively).

For these communities, the main demand barriers leading to ‘Vaccine Hesitancy’ were tackled mainly through (i) targeted messaging and (ii) community engagement.

##### (i) Targeted messaging

Weekly collected uptake data by vaccine cohort and ethnicity were used to target messaging, which was found to improve uptake. Information was translated to relevant languages and distributed to residents in low uptake groups. In consultation with residents, information was also tailored to focus on reported concerns, hence centring on improving trust and correcting erroneous beliefs about vaccine effectiveness and countering confusion on side effects. Common approaches to engaging with communities included Question and Answer sessions, Frequently Asked Questions and Webinars (*n* = 27 LAs). Live streaming and out-of-hours sessions were provided to enable shift workers to attend. Health care staff had reported that they particularly valued a ‘peer champion approach’ involving one-to-one supportive conversations (*n* = 27 LAs) in which vaccine concerns could be discussed in a safe space. This avoided potential barriers associated with the power dynamics inherent in relationships with line managers.

##### (ii) Community engagement

Engagement by community champions or social prescribers (*n* = 21 LAs) was found valuable by 10 LAs. For example, vaccination officers and community-based health care workers, often from the same communities as those targeted, acted as ‘bridges’ between residents and provider organizations to address hesitancy.

Digital/social media was used by 24 LAs, including videos of trusted community leaders receiving the vaccine and addressing mistrust issues. Traditional media were also adopted (*n* = 19 LAs), whereby local newspapers included NHS-information translated for specific communities (e.g. Turkish or Kurdish).

In addition, focus groups (*n* = 10 LAs), often organized in community venues used by ethnic minority groups, were perceived to be ‘effective in getting the message out generally and provided a forum for communities, using trusted [community] leaders’ (LA respondent). These addressed topics of particular relevance, such as structural inequalities and racism.

#### Future lessons

The national imperative to vaccinate as many people in each cohort as quickly as possible came at the expense of addressing trust and hence improving equity in uptake:

‘This is very much a nationally driven programme and it doesn’t work locally. You need local figures, local influence, understanding of the local situation, and then you need to flex your programme locally.’ (LA respondent)

Some local relationships were reportedly established after uptake inequalities had manifested. Instead, respondents argued that:

‘[y]ou need to have ongoing engagement with communities, identifying their concerns and addressing them on an iterative basis’ (LA respondent)

The ICS structure was deemed by some LAs to be too bureaucratic:

‘We need more autonomy for local authorities and genuine partnership with the acute trusts. The ICS structure has been a real barrier.’ (LA respondent)

Fruitful collaborations were also stymied by unhelpful emphasis on borough boundaries:

‘We need to start thinking about the artificial nature of borough boundaries, which contain shared communities who don’t always have access to the same treatment’ (LA respondent)

### *Vaccine supply (access*)

#### Barriers

The booking of appointments was reportedly problematic (*n* = 11 LAs). Specific issues included delays in general practices providing information on eligibility, leading residents to book vaccinations before their cohorts had officially opened (Fig. 1). Difficulties were also reported with navigating on-line booking systems, reliance on a working phone number and language barriers.

The location of vaccination centres also raised access barriers (*n* = 7 LAs) particularly earlier in the vaccination programme when mass (larger/centralized) centres were common with fewer local alternatives. This barrier remained to some extent, even when local centres opened, as appropriate storage requirements resulted in the restriction of some vaccines to mass centres (*n* = 9 LAs).

Limited opening hours (*n* = 2 LAs) also reduced access for shift workers.

#### Interventions

**Fig. 4:**
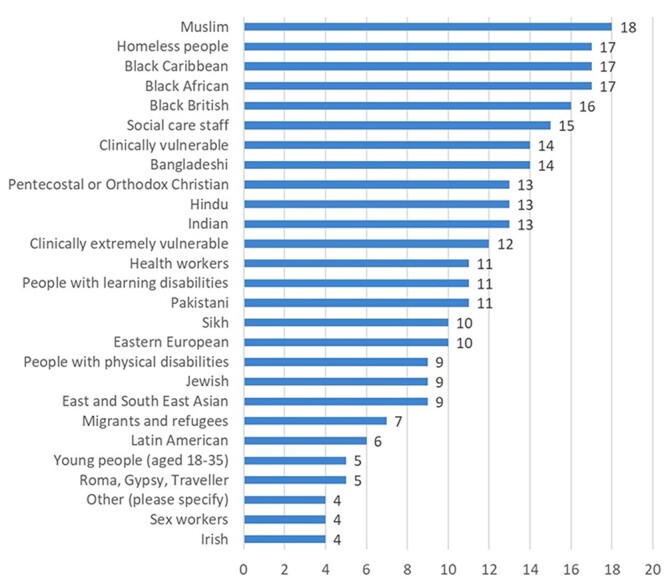
Number of LAs (*n* = 32) reporting activities to address supply side barriers (‘Access’) among specific communities.

A diversity of communities were targeted by supply side interventions to improve access (Fig. 4).

All system representatives discussed the importance of the location of vaccination sites. While larger centralized centres and ‘surge events’ (i.e. vaccination at scheduled times in landmark venues such as sports stadiums) reportedly reached overall larger numbers of residents, it was argued that a ‘hyper-local’ approach reached communities that otherwise might not get vaccinated. This approach determined vaccination location using distance to transport links and ‘real-time’ data on geographical areas with lowest uptake. Sites included temporary ‘pop-up’ clinics, vaccination in pharmacies and other community venues or outreach to ‘housebound’ or homeless populations. These initiatives were promoted by local representatives familiar to residents and these allowed vaccinations to take place in ‘moment[s] of engagement’ (LA respondent). For example, community church pop-up sites reportedly delivered vaccination to >60% Black attendees in one LA.

Composite invitation/booking approaches (*n* = 22 LAs) such as ‘a letter, text message, email and phone call … ensure[d] that all residents [stood] the best possible chance of being contacted’ (LA respondent).

#### Future lessons

Appropriate placement of sites and establishment of outreach initiatives required collaboration:

‘From early on we had an understanding that no single organisation can deliver something like this – it relies on collaboration, in this case across four agencies and at multiple other levels’ (‘Regional’ (Supplementary Material 1) respondent)

However, even for organizations with less tradition of collaboration, the unprecedented situation of the pandemic and urgent need to roll out the vaccine instilled a common purpose:

‘This was an example of a sector-wide intervention which could be replicated, where the relationships were key. It took a fairly organic form, but was successful in breaking down organisational barriers through a common purpose.’ (ICS respondent)

It was suggested that this experience should be integrated into standard practice:

‘Build on the relationships that now exist by ensuring that collaborations formed now could be used to address other health needs such as relationship with NHS and local authorities.’ (LA respondent)

## Discussion

### Main finding of this study

We report on work undertaken in a large metropolitan city, London, to increase COVID-19 vaccine uptake. We demonstrate stark differences in uptake from 90% in White British compared to 57% in Black ethnic groups after the first 6 months of vaccination. Mistrust in government institutions and information provided, together with access barriers, were reported to drive such inequalities.

### What is already known on this topic

National evidence confirms the lower COVID-19 vaccine uptake in ethnic minority, and particularly in Black ethnic groups, compared to the White majority.^18^ Some evidence suggests inequality gaps may decrease slightly over time, although higher rates remain among White British people.^18^ We also acknowledge the relevance of some evidence on vaccination drives that predate the mass roll-out of the COVID-19 vaccines. Our findings that lower vaccine uptake among certain ethnic groups stems from ‘racism and historic injustices’ align with pre-existing international literature which argues that an understanding of hesitancy requires consideration of the ‘historical, political and socio-cultural context in which vaccination occurs’.^3^ Our results also concur with other studies reporting that centralized, mass vaccination centres can be challenging to access and may hence increase health inequalities,^19^ while community outreach activities promote engagement.^20^^,^^21^

### What this study adds

To our knowledge, our study provides the most comprehensive quantitative analysis combined with an in-depth exploration of the early stages of COVID-19 vaccination in London, mapping inequalities, probing uptake barriers and identifying interventions to address these.

Crucially, although lack of trust and the perceived costs of vaccination may be common throughout the country, adequately supported ‘hyper-local’, responsive interventions, which were promptly mobilized and unconstrained by administrative boundaries, were viewed as key to overcoming London residents’ concerns. Initiatives need to be multi-faceted and flexible, co-designed and delivered by those communities whose beliefs and needs inform both vaccine hesitancy and access. One-to-one workplace conversations, out-of-hours Question and Answer sessions, support from trusted community champions and newspapers and pop-up and outreach vaccination models are examples of the intensive interventions required. Conversely, reliance on single-component ‘top-down’ interventions may widen inequalities, further embed mistrust and are unlikely to succeed.

Nevertheless, deeply embedded distrust of governmental provider organizations takes time and resources to address via in-depth understanding of communities’ perspectives. Long-term investment in regional and local partnerships and community engagement also in times outside of public health emergencies are thus essential.

These partnerships can be sustained through clear articulation of common goals. The wider organizational culture literature^22^ and a recent UK government report^23^ demonstrate how a shared, common purpose and collective sense of urgency facilitates collaboration across different levels. Our study demonstrates the importance of this even in the complex, multi-layered network of London, including multiple regional bodies and local actors, both statutory (NHS, LAs) and voluntary (community organizations), which prior to the COVID-19 pandemic pursued priorities which often did not align.

### Limitations of this study

Our study provides generalisable insights for enhancing uptake and reducing inequalities during current and future vaccination programmes in the UK and similar countries that have experienced low uptake, e.g. Australia.^24^ However, we report four limitations. Firstly, all qualitative data were reported by system leaders/public health staff and not from LA residents themselves. Secondly, the data cut-off point of 6 June 2021 meant we cannot provide full understanding of barriers and successful interventions in younger cohorts. Thirdly, for factors reported as successful in improving uptake, we did not have ‘real-time’ data of the establishment of these interventions to enable correlation with changes in uptake. Finally, we did not control for likely confounders such as socio-demographic disadvantage (often reported by area-level, e.g. Index of Multiple Deprivation, rather than matched with individual data). However, UK surveys conducted during the pandemic but before COVID-19 vaccine roll-out highlighted that beliefs about the virus and vaccines are stronger determinants of willingness to accept a vaccine than sociodemographic characteristics.^25^^,^^26^ This aligns with our reports of the detrimental impact of mistrust.

## Conclusion

Our detailed findings can be used to establish a framework for sustained, trusted engagement with communities across complex, large cities in preparation for future public health emergencies.

## Supplementary Material

Supplementary_Material_1_-_Overview_of_the_vaccine_delivery_programme_in_England_fdac038

Supplementary_Material_2_-_Survey_questions_fdac038

Supplementary_Material_3_-_Interview_guide_fdac038

## Data Availability

The quantitative data underlying this article were provided by NHS and permission needs to be sought for any sharing of this data. The article’s other data are available in the article or its online supplementary material, or may be shared on reasonable request to the corresponding author.

## Notes

**Kristoffer Halvorsrud,** Research Fellow

**Jenny Shand,** Director of Strategy and Partnerships and Associate Professor

**Leonora G. Weil,** Public Health Consultant

**Andrew Hutchings,** Assistant Professor

**Ana Zuriaga,** COVID-19 Response Analyst

**Dane Satterthwaite,** London Region Head of PHM (Acting) and COVID-19 Vaccine Programme-Inequalities Analytics

**Jennifer L.Y. Yip,** Consultant in Public Health

**Cyril Eshareturi,** Senior Programme Manager

**Julie Billett,** Director of Operations, London

**Ann Hepworth,** Director of System Improvement

**Rakesh Dodhia,** Technical Consultant

## Notes

**Ellen C. Schwartz,** Immunisation Lead

**Rachel Penniston,** Head of Improvement

**Emma Mordaunt,** Improvement Projects Manager

**Sophie Bulmer,** Network Development Lead

**Helen Barratt,** Clinical Senior Research Associate/Hon Consultant in Public Health

**John Illingworth,** Evaluation Lead and Patient Safety Advisor

**Joanna Inskip,** Briefings and Engagement Manager

**Fran Bury,** Public Health Registrar

**Deborah Jenkins,** Public Health Registrar

**Sandra Mounier-Jack,** Associate Professor

**Rosalind Raine,** Professor
