# Correction to: Tackling barriers to COVID-19 vaccine uptake in London: a mixed-methods evaluation

**DOI:** 10.1093/pubmed/fdac059

**Published:** 2022-05-11

**Authors:** Kristoffer Halvorsrud, Jenny Shand, Leonora G Weil, Andrew Hutchings, Ana Zuriaga, Dane Satterthwaite, Jennifer L Y Yip, Cyril Eshareturi, Julie Billett, Ann Hepworth, Rakesh Dodhia, Ellen C Schwartz, Rachel Penniston, Emma Mordaunt, Sophie Bulmer, Helen Barratt, John Illingworth, Joanna Inskip, Fran Bury, Deborah Jenkins, Sandra Mounier-Jack, Rosalind Raine

**Affiliations:** Department of Applied Health Research, University College London (UCL), London WC1E 7HB, UK; UCLPartners, London W1T 7HA, UK; Department of Clinical, Education & Health Psychology, UCL, London WC1E 6BT, UK; UK Health Security Agency, London SE1 8UG, UK; Department of Health Services Research and Policy, London School of Hygiene and Tropical Medicine, London WC1E 7HT, UK; UK Health Security Agency, London SE1 8UG, UK; NHS England and NHS Improvement London, London SE1 6LH, UK; Office for Health Improvement and Disparities, London region, London SW1H 0EU, UK; Public Health England London, London SE1 8UG, UK; Office for Health Improvement and Disparities, London region, London SW1H 0EU, UK; NHS England and NHS Improvement London, London SE1 6LH, UK; NHS England London Shared Service, London SE1 6LH, UK; Association of Directors of Public Health, London EC4Y 0HA, UK; UCLPartners, London W1T 7HA, UK; UCLPartners, London W1T 7HA, UK; UCLPartners, London W1T 7HA, UK; Department of Applied Health Research, University College London (UCL), London WC1E 7HB, UK; UCLPartners, London W1T 7HA, UK; Office for Health Improvement and Disparities, London region, London SW1H 0EU, UK; NHS England and NHS Improvement London, London SE1 6LH, UK; Royal Free London NHS Foundation Trust, London NW3 2QG, UK; Department of Global Health and Development, London School of Hygiene and Tropical Medicine, London WC1E 7HT, UK; Department of Applied Health Research, University College London (UCL), London WC1E 7HB, UK

In the originally published version of this manuscript, the following authors were identified as affiliated to UK Health Security Agency in error: Jennifer L Y Yip, Cyril Eshareturi, Julie Billett, and Joanna Inskip.

These errors have been corrected and, where necessary, the correct affiliation added instead.

