# Supplementary material for: Tackling barriers to COVID-19 vaccine uptake in London: a mixed-methods evaluation
Source: J Public Health (Oxf). 2022 Apr 4;45(2):393–401. doi: 10.1093/pubmed/fdac038 (PMC8992332; doi:10.1093/pubmed/fdac038)
Supplement: Supplementary_Material_1_-_Overview_of_the_vaccine_delivery_programme_in_England_fdac038 [file supplementary_material_1_-_overview_of_the_vaccine_delivery_programme_in_england_fdac038.docx]

**Supplementary Material 1:**

Overview of the vaccine delivery programme in England

Joint Committee on Vaccination and Immunisation (JCVI) vaccination priority cohorts[1, 14]

**Cohort 1:** Care home residents, residential workers

**Cohort 2:** General population aged 80+ years, National Health Service (NHS) health care workers, social care workers

**Cohort 3:** General population aged 75-79 years

**Cohort 4:** General population aged 70-74 years, Clinically Extremely Vulnerable

**Cohort 5:** General population aged 65-69 years

**Cohort 6:** ‘At risk’, care home residents – younger adults, Department for Work and Pensions

(DWP) carers, Local Authority (LA) carers, learning disability register, QCovid risk assessment

**Cohort 7:** General population aged 60-64 years

**Cohort 8:** General population aged 55-59 years

**Cohort 9:** General population aged 50-54 years

**Cohort 10:** General population aged 40-49 years

**Cohort 11:** General population aged 30-39 years

**Cohort 12:** General population aged 18-29 years

Main delivery channels in London[1, 15-18]

London consists of five Integrated Care Systems (ICS) – the organisational health and care partnerships that meet the healthcare needs in South East, South West, North East, North West and North Central London, respectively, and which between them cover London’s 32 local authorities (LAs). The planning and operational delivery of COVID vaccination in London at that time was led by these five ICSs, supported by the NHS England and Improvement Vaccine Delivery Programme*,* Public Health England (PHE) London Region, Greater London Authority, London authorities and community champion leads. London’s individual LAs were granted flexibility to adapt approaches to delivery and access to address their local needs.

The nationally mandated approach in England to COVID-19 vaccine delivery is available through different channels:

- an appointment could be booked online for those with an NHS number for a vaccine at mass vaccination centres including sites set up in larger venues such as sports stadiums and hospital hubs, or more local vaccination service sites (e.g. general practices (GPs) or pharmacies);
- a walk-in vaccination site may be accessed without a prior appointment, providing an alternative without needing to be registered with a GP or have a proof of address;
- outreach and roving models of delivery to reach populations unable or less likely to engage with these main routes.
