# Supplementary material for: Tackling barriers to COVID-19 vaccine uptake in London: a mixed-methods evaluation
Source: J Public Health (Oxf). 2022 Apr 4;45(2):393–401. doi: 10.1093/pubmed/fdac038 (PMC8992332; doi:10.1093/pubmed/fdac038)
Supplement: Supplementary_Material_2_-_Survey_questions_fdac038 [file supplementary_material_2_-_survey_questions_fdac038.docx]

**Supplementary Material 2:**

Survey questions

| What type of interventions have you put in place to improve COVID vaccine uptake in your area? | Digital/social media |
| --- | --- |
|  | Traditional media |
|  | Focus groups to ascertain views of the population |
|  | Community champions/social prescribers |
|  | Question and Answer (Q & A) sessions or webinars with experts |
|  | One-to-one supportive conversations |
|  | Other outreach activities (please specify) |
| Please provide a description of activities for each type of intervention with as much detail as possible. It would be particularly helpful to understand which communities or population groups were the intended audience for these activities. | Digital/social media |
|  | Traditional media |
|  | Focus groups to ascertain views of the population |
|  | Community champions/social prescribers |
|  | Question and Answer (Q & A) sessions or webinars with experts |
|  | One-to-one supportive conversations |
|  | Other outreach activities (please specify) |
| Did these interventions target any specific communities or population groups? Please tick all that apply. | Black African |
|  | Black Caribbean |
|  | Black British |
|  | Indian |
|  | Pakistani |
|  | Bangladeshi |
|  | East and South East Asian |
|  | Latin American |
|  | Eastern European |
|  | Roma, Gypsy, Traveller |
|  | Irish |
|  | Migrants and refugees |
|  | Muslim |
|  | Jewish |
|  | Hindu |
|  | Sikh |
|  | Pentecostal or Orthodox Christian |
|  | People with physical disabilities |
|  | People with learning disabilities |
|  | Health workers |
|  | Social care staff |
|  | Sex workers |
|  | Homeless people |
|  | Young people (aged 18-35) |
|  | Clinically vulnerable |
|  | Clinically extremely vulnerable |
|  | Other (please specify) |
| If not already described, please outline any specific work you have done with any of these communities or population groups | Open-Ended Response |
| How have you addressed the following elements of service delivery to improve vaccination uptake? Please provide an example of what was done for each element. It would be particularly helpful to understand if these were undertaken for specific communities or population groups. | The location of the vaccination centres (for example proximity to transport links for accessibility) |
|  | Type of vaccination centres (mass centre, general practitioner (GP)-led, community pharmacy, cultural or community centres, etc.) |
|  | Timing of appointments (for example the provision of out-of-hours appointments) |
|  | Design and application of invitation/booking system (for example providing letter, text or phone call invitations) |
|  | Re-scheduling (for example repeat calls or messages of those who missed or did not accept their first-time invitations) |
|  | Follow up of did not attends (DNAs) |
|  | Others (please describe): |
| Did these elements of service delivery accommodate the needs of any of the following specific communities or population groups? Please tick all that apply. | Black African |
|  | Black Caribbean |
|  | Black British |
|  | Indian |
|  | Pakistani |
|  | Bangladeshi |
|  | East and South East Asian |
|  | Latin American |
|  | Eastern European |
|  | Roma, Gypsy, Traveller |
|  | Irish |
|  | Migrants and refugees |
|  | Muslim |
|  | Jewish |
|  | Hindu |
|  | Sikh |
|  | Pentecostal or Orthodox Christian |
|  | People with physical disabilities |
|  | People with learning disabilities |
|  | Health workers |
|  | Social care staff |
|  | Sex workers |
|  | Homeless people |
|  | Young adults (aged 18-35) |
|  | Clinically vulnerable |
|  | Clinically extremely vulnerable |
|  | Other (please specify) |
| If not already described, please outline how these elements of service delivery accommodated the needs of the specific communities or population groups highlighted/ticked above. | Open-Ended Response |
| Please describe barriers that you have encountered to COVID vaccine uptake within your local borough. Barriers may include vaccine hesitancy, access barriers or other reasons for delayed uptake in different cohorts. You may also want to consider barriers at different levels of the system, including borough-wide, community, and individual levels. | Open-Ended Response |
| What work have you done to better understand these barriers to COVID-19 vaccine uptake in your population? | Open-Ended Response |
| Has your organisation assigned dedicated resource locally to deliver activities to increase vaccination uptake? Please describe. | Open-Ended Response |
| What governance arrangements are in place locally to oversee work to increase vaccine uptake and address inequalities? | Open-Ended Response |
| How have you worked with partners across organisations to increase vaccine uptake in your borough? | Open-Ended Response |
| What evaluations (or measurement of impact) have you done of the interventions you have described in the previous questions? When answering this question, please could you also indicate:   whether it was for a single programme (please define) or for your work overall; whether you worked with an academic partner; what data were collected, for example routine and/or specially collected quantitative and/or interview or other qualitative data. | Open-Ended Response |
| What are the key lessons you have learnt from addressing COVID vaccine hesitancy and improving COVID vaccine uptake? For example, what would you do differently in the future? | Open-Ended Response |
| What have been your key areas of success? Please give examples of how the interventions have improved COVID vaccination uptake. | Open-Ended Response |
| What additional support would you like from regional teams?  For example additional data, toolkits, updates, or provision of specific resources. Please also include additional barriers you have faced where you require support from elsewhere in the system (eg regional or national teams to resolve it). | Open-Ended Response |
