# Supplementary material for: Tackling barriers to COVID-19 vaccine uptake in London: a mixed-methods evaluation
Source: J Public Health (Oxf). 2022 Apr 4;45(2):393–401. doi: 10.1093/pubmed/fdac038 (PMC8992332; doi:10.1093/pubmed/fdac038)
Supplement: Supplementary_Material_3_-_Interview_guide_fdac038 [file supplementary_material_3_-_interview_guide_fdac038.docx]

**Supplementary Material 3:**

Semi-structured interview guide and questions

2 June 2021

**Introduction**

Thank you for taking time to talk to us about your experience of rolling out the COVID-19 vaccination. We would like to ask for your personal reflections of the programme to date. The questions are divided into three sections:

1. Demand – reflections on activity to increase uptake of the vaccine and address hesitancy.

2. Supply – reflections on activity to make the vaccine more easily accessible to people that wanted to have it.

3. Legacy – reflections on how we can take the lessons and apply them to the future, across the NHS, social care and council activities.

Interviews will be analysed and written into a report, shared back with interviewees and the wider system. This is also part of an academic evaluation which will be published. We would like to acknowledge you as one of the contributors, would you be happy for us to do this?

**Consent**

Remind the interviewee that the interview will be transcribed (and recorded if interviewer wishes).

Inform them that quotes will not be attributed beyond Region / ICS / Borough level. their acknowledgment in the formal report will not detail their involvement.

**Interviewee information**

Please confirm the interviewee’s details if you do not already have the following:

Name, role (original and COVID specific), organisation, geography (eg., London region, North East London ICS, or Bexley), date of interview

**QUESTIONS**

**Demand**

1. What has **worked well** with regards to interventions that you have put in place to increase uptake of the COVID-19 vaccine or address hesitancy?
   Prompt: What surprised you?
2. Were there things you did which **did not work well** with regards to increasing uptake of the vaccine and addressing hesitancy that you would avoid in the future?

Prompt: Is this specific to a population group?

1. Based on your experience of rolling out the COVID-19 vaccine to date, what are your **recommendations for the future** with regards to increasing uptake and addressing hesitancy for:
2. Maximising uptake for surge vaccination and remaining cohorts (and for potential boosters)? PROMPT: what barriers and facilitators would you expect to encounter?
3. Maximising uptake of future routine vaccinations?
4. Future pandemics?

**Supply**

1. What has **worked well** with regards to interventions that you have put in place to increase access to a COVID-19 vaccine?
   Prompt: What surprised you?
2. Were there things you did that **did not work well** with regards to making the vaccine more accessible to people that you would avoid in the future?

Prompt: Is this specific to a population group?

1. Based on your experience of rolling out the COVID-19 vaccine to date, what are your **recommendations for the future** with regards to increasing access to the vaccine for:
2. Maximising uptake for **surge vaccination** and remaining cohorts (and for future boosters)? PROMPT: what barriers and facilitators would you expect to encounter?
3. Maximising uptake of future routine vaccinations?
4. Future pandemics?

**Legacy**

1. As we move to consider the legacy of the vaccine programme to date, what have we learnt about each of the following domains that can help shape the way we work or what we do in the future?
2. **Inequalities –** How we reduce existing inequalities and mitigate against creating new ones?
   (Guidance for interviewer: if appropriate, make sure we know which group the respondent is referring to)
3. **Infrastructure** – What we have put in place to deliver the vaccines, and what lessons we can extract for routine vaccine programmes, screening programmes and/or diagnosis pathways?
4. **Workforce** - Our relationship with our staff and how we work with them?
5. **Partnerships** – For example between the NHS and local government, with and between health and care providers, and with community groups?
6. **Community engagement** - Our relationship with the community, how to access different groups and generate two-way dialogues?
   Prompt: how will you sustain this engagement?
7. Are there any further reflections you would like to add?

Thank you for taking part in this interview, we really appreciate your time and will make sure to share the outputs with you.
